# Novel risk factors associated with fatal musculoskeletal injury in Thoroughbreds in North American racing (2009–2023)

**DOI:** 10.1111/evj.14503

**Published:** 2025-03-25

**Authors:** Euan David Bennet, Tim D. H. Parkin

**Affiliations:** ^1^ School of Biodiversity, One Health, and Veterinary Medicine University of Glasgow Glasgow UK; ^2^ Bristol Veterinary School University of Bristol Bristol UK

**Keywords:** fatal injury, horse, horse racing, musculoskeletal, risk factors, Thoroughbred racing

## Abstract

**Background:**

The Equine Injury Database (EID) is a census‐level record of Thoroughbred racing in North America, currently recording 95.6% of all race starts in 2023, along with partial training and veterinary histories of each horse.

**Objectives:**

To identify horse‐, race‐ and track‐level risk factors associated with race‐related fatal musculoskeletal injury (MSI) of Thoroughbred racehorses in North America.

**Study Design:**

Retrospective cohort study.

**Methods:**

The study cohort included all race starts made by horses born after 31 December 2006, at tracks that fully report to the EID and consisted of 3,851,659 race starts made by 250,840 Thoroughbred racehorses (median [IQR] starts per horse 11 [5–22]) at 115 racetracks in the USA and Canada between 2009 and 2023, inclusive. Ninety‐seven potential risk factors were investigated using univariable and multivariable logistic regression modelling.

**Results:**

Exactly 5733 fatal MSIs were recorded, an incidence of 1.49 fatal MSIs per 1000 starts. Twenty risk factors had statistically significant associations with increased or decreased odds of fatal MSI. Previously unidentified risk factors included claiming race‐related variables and void claim rules (VCR). Horses racing as claimers were at increased odds compared with those who were not (odds ratio 1.31, 95% confidence interval 1.19–1.45, *p* < 0.001 for the lowest claim prices). Starts in races with the strictest VCR were at reduced odds compared with starts in races with no VCR (OR 0.76 [0.67–0.85], *p* < 0.001).

**Main Limitations:**

Availability of new data sources increased substantially during the 15‐year time period of the study, meaning some new risk factors are limited in scope compared with others.

**Conclusions:**

Thoughtful integration of new data sources with race‐level data can lead to new insights into risk factors for deleterious outcomes affecting racehorses. Results can inform ongoing efforts to mitigate the risk of fatal MSI, through direct regulatory intervention and through building a risk profile based on individual history and track‐level factors.

## 1 INTRODUCTION

Thoroughbred racing has been a primary focus of equine sports research over the past few decades. Risk factors associated with horse fatalities have been a common topic explored in the literature. The most common cause of exercise‐related Thoroughbred fatality is death or euthanasia as a result of fatal (sometimes described as catastrophic) musculoskeletal injury (fatal MSI).^1^, ^2^, ^3^, ^4^, ^5^, ^6^, ^7^, ^8^, ^9^, ^10^, ^11^, ^12^, ^13^, ^14^, ^15^, ^16^, ^17^, ^18^, ^19^, ^20^, ^21^, ^22^, ^23^, ^24^, ^25^, ^26^, ^27^, ^28^, ^29^, ^30^, ^31^, ^32^, ^33^ The other type of fatality typically recorded is sudden death, which is a much rarer outcome.^34^, ^35^, ^36^, ^37^, ^38^, ^39^ Consistently, risk factors for fatal MSI have been identified from multiple domains, including exercise/veterinary history of the horse, along with race‐level factors such as surface and distance. Hitchens et al. (2019) provide a comprehensive meta‐analysis of risk factors across all studies.^29^

The Equine Injury Database (EID) was established in 2009 by The Jockey Club as a comprehensive record of injuries in Thoroughbred racing in the United States and Canada.^40^ Between 2009 and 2023 inclusive, the EID recorded injuries in 92.3% of race starts made at 163 racetracks in the two countries. The proportion of fully reported race starts has increased during the time period, and in 2023, the EID recorded injuries in 95.6% of all race starts. Annual monitoring of horse fatality statistics in the EID has reported a sustained decrease in the incidence of race‐related horse fatalities from all causes since 2009.^41^

The most recent large‐scale study of the EID was published in 2016 and covered the period 2009–2013.^12^ Since the publication of that study, the EID has expanded to include new data sources as well as millions more recorded race starts, resulting in a dataset with greater breadth and depth than that which was previously available. There have also been a number of regulatory and management changes introduced over the last decade that warrant a new analysis of risk factors for fatal MSI.

The present study was a retrospective cohort study that aimed to identify risk factors associated with race‐related fatal MSI in Thoroughbred flat racing in the USA and Canada between 2009 and 2023, inclusive. The primary hypothesis was that some combination of risk factors at the level of the horse, trainer, race and track—including factors that have not previously been investigated for this study cohort—would be associated with the risk of a horse experiencing a fatal MSI within 3 days of racing.

**FIGURE 1:**
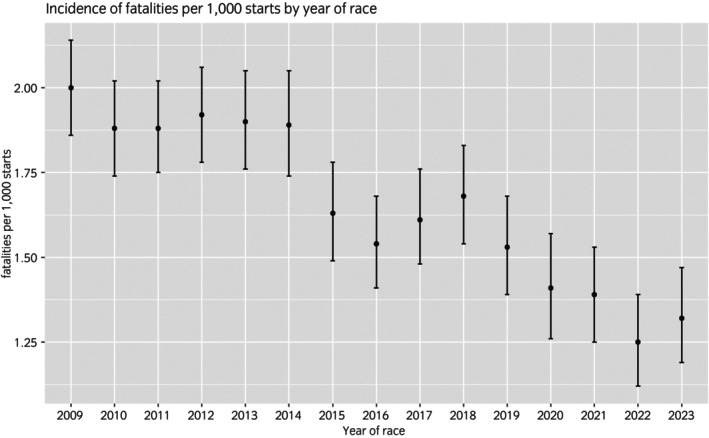
Incidence of racehorse fatalities by all causes within 3 days of race date per 1000 starts at fully reporting tracks in each year of the Equine Injury Database (EID), as reported by the Jockey Club.^41^ Error bars show the 95% confidence intervals calculated using the Wilson score interval.^54^

Figure 1 was adapted from data in the annual audit published by The Jockey Club in 2024,^41^ and shows the incidence of fatalities per 1000 starts in each year for all race starts made at tracks that fully reported to the EID from 2009 to 2023. An overall trend of decreasing incidence is apparent from 2015 to 2023. The year of the race was excluded from the list of potential risk factors in this study: the underlying cause of any association between year and fatal MSI is undoubtedly driven by a combination of risk factors that have themselves changed over time, many of which were available for investigation in the present study.

Many potential risk factors are likely to be related to, or influenced by, regulations governing the sport. During most of the existence of the EID, regulatory change in the sport was applied through rules changes in every individual jurisdiction for Thoroughbred racing. In the United States, each state is a single jurisdiction. For example, the National Uniform Medication Program proposed in 2013 was adopted gradually during 2013 and 2014.^42^ As such, even regulatory changes that were adopted within all jurisdictions pre‐2022 should be viewed as an ongoing process as opposed to a single event. Hence it is challenging to identify specific interventions as being associated with year‐to‐year changes in incidence of fatal MSI. It is more likely that ongoing work from stakeholders in the industry, who have continually engaged with the evidence provided through analysis of the EID and other data, has led to a large number of small reductions in risk across multiple individual risk factors. This so‐called ‘aggregation of marginal gains’ is evident from the overall trend shown in Figure 1. The most likely explanation of, for example, the statistically significant decrease in incidence from 2014 to 2015 (Chi‐squared test *p* < 0.001) is that this was the result of this marginal gains approach reaching fruition across multiple regulations, policies and risk factors.

In December 2020, the United States Congress passed the Horseracing Integrity and Safety Act, resulting in the creation of the Horseracing Integrity and Safety Authority (HISA).^43^, ^44^, ^45^ The first HISA racetrack safety regulations were enforced from July 2022. This marked the first time such a regulatory body had simultaneously legislated across all participating state‐level racing jurisdictions in the United States. 71.5% of race starts recorded in the EID during 2023 were made under HISA regulations. It should, however, be noted that it remains the case that not all state‐level racing jurisdictions currently participate in HISA.

The Veterinarian's List (often referred to as the ‘vet list’) is an ongoing register of horses deemed ineligible to race. Reasons to be placed on the vet list include if the horse is medically compromised due to illness or injury, lame, unsound, or physically distressed. Once a horse is added to the vet list, it remains on it for a period specified by the racing regulatory veterinarian, and horses must pass another vet examination before they are removed from the vet list and permitted to race again.

Claiming races are a common feature of Thoroughbred racing in North America and refer to a situation where some or all of the horses participating in the race are available for sale before the race. Each horse has an advertised claim price and can be ‘claimed’ (purchased) until shortly before the race begins with ownership of the horse usually transferring after the race has completed. In recent years, a range of so‐called ‘void claim rules’ have been introduced in some jurisdictions, specifying circumstances in which any claims made before a race must be cancelled (voided), depending on the horse's condition at the end of the race. In the United States, four types of void claim rules were applied in different states at different times. A fifth type—the strictest of all types—was introduced through HISA. Rule 2262 of the HISA regulations states that any claims are voided if the horse dies or is euthanised, is vanned off the track, bleeds, or tests positive for any banned substance. Furthermore, all claimed horses must be examined by a regulatory veterinarian following the race, and claims are voided if they are added to the vet list.^45^, ^46^ Table 1A summarises the five different void claim rules that were present during the period covered by the study.

**TABLE 1:** Summary of definitions of void claim rules (Table 1A) and triage scores (Table 1B).

(A)	
Void claim rule	Outcomes that void a claim
Type 1	Any fatality only
Type 2	Any fatality, or horse added to vet list
Type 3	Any fatality, horse added to vet list, or bleeds
Type 4	Any fatality, or horse is vanned off the track
Type 5 (HISA)	Any fatality, horse is vanned off the track, bleeds, tests positive for any banned substance, or is added to the vet list

(B)	
Triage score	Description on injury reporting form
0	No lameness detected
I	Grade III or lower lameness; no obvious limb deformity
II	>Grade III lameness; no obvious limb deformity
III	Mild to moderate limb instability in 1 plane; closed injury
IV	Severe instability in 1 plane; closed injury
V	Limb instability in 2 or more planes; loss of column of support (open or closed); open injury; fracture/joint capsule/tendon sheath

*Note*: Horses are added to the vet list if the regulatory veterinarian at the race observes that they are medically compromised due to illness or injury, lame, unsound, or physically distressed. Usually, a triage score greater than 0 would also require a horse to be added to the vet list.

The EID uses a standardised form with which a reporting veterinarian at each race can record any pre‐ or post‐race injuries observed in racehorses. The form includes a ‘triage score’ which is assigned if a horse exhibits lameness, limb deformity, or limb instability. Note that a triage score greater than zero would usually also result in the horse being added to the vet list. Table 1B summarises the levels of triage score that are collected in the EID.

## 2 MATERIALS AND METHODS

The unit of observation for this study was the race start, which was defined as one horse starting in one race. The full EID from 2009 to 2023 contains detailed information about 5,102,473 race starts, made by 324,407 individual horses at 163 racetracks in the United States and Canada. The study cohort was selected from the full EID using the following inclusion/exclusion criteria:

1. 395,258 race starts at non‐reporting tracks were excluded from the study cohort. Only race starts made at tracks that were fully reporting to the EID at the time of the race were included.
2. 854,785 race starts made by any horse born before 1 January 2007 was excluded. This ensured that the study cohort included every race start of each individual horse's racing career, up to 31 December 2023.
3. 462 race starts for which an outcome of ‘sudden death’ was recorded were excluded from the study cohort.
4. 259 fatal MSI outcomes which were recorded as having occurred more than 3 days after the race date were excluded from the study cohort.

The final study cohort included 3,851,659 race starts, made by 250,840 individual Thoroughbred racehorses at 115 racetracks which fully reported to the EID. The case definition used was any racing MSI resulting in either death or euthanasia, with fatality being recorded within 3 days of race day. A total of 5733 cases were present in the final study cohort; the remaining 3,845,926 race starts were included as controls.

The EID is updated annually, and in its unprocessed form, contains a detailed record of each race start. New potential risk factors were created by mining the recorded variables to extract, for example, individual racing histories of horses. Other data sources—initially separate from the main EID, but also recorded by the Jockey Club—were reconciled to the racing data during this study. Four primary data sources were added: (1) the vet list; (2) partial training records; (3) records of horses put up for sale early in their lives; and (4) records of void claim rules and their dates of enforcement at each track. Note that data reconciled with the main EID were fully recorded and covered the entire study period 2009–2023. Very few variables selected for analysis had any missing data: for example, the date of the horse's previous workout was unavailable for 1632 race starts (0.04% of all starts). These observations were grouped into the reference level of the categorical form of the variable during testing at the univariable modelling stage. The recorded speed was unavailable for 1439 starts (0.04% of all starts)—various forms of variables involving speed were examined, but none were retained during final model building.

After reconciliation of these data sources, the full database consisted of 218 variables relating to the 3,851,659 observations in the study cohort. A total of 97 potential risk factors were identified as available for modelling—a description of these can be found in Table S1. This initial phase of risk factor identification was informed by previous studies, data availability and biological plausibility.

A multivariable logistic regression model was constructed to investigate associations between potential risk factors and fatal MSI. R version 4.4.0 (R Foundation for Statistical Computing) and the Tidyverse package^47^ were used for all data processing and modelling. During the first stage of model building, univariable logistic regression was applied to each potential risk factor in turn. Factors found to be associated with fatal MSI with a statistical significance at the 80% level (*p* < 0.2) became candidates for the final multivariable model. Risk factors in continuous form were assessed in both continuous and transformed form—including categorical forms such as quartiles—to identify the form that produced the best model fit, according to the Akaike Information Criterion (AIC).^48^, ^49^ Table S1 describes the chosen categorisation for each risk factor.

The final multivariable logistic regression model was built using a stepwise backwards‐removing process; the model with the lowest AIC value was selected at each iteration. Model validation included assessing biologically plausible interaction terms and testing for confounding between variables rejected at any stage and those retained in the final model.^49^ To assess for potential clustering, three mixed‐effects logistic regression models were tested, consisting of the final fixed‐effects model with horse, trainer and track included as random effects. Goodness of fit of the final model was assessed using the Hosmer–Lemeshow test.^50^ Post hoc power calculations showed that for continuous variables, the final model had at least 80% power to detect odds ratios of 1.04 or above with 95% confidence. For binary categorical variables, the odds ratio threshold was 1.08.

## 3 RESULTS

The study cohort consisted of 3,851,659 race starts, of which 5733 resulted in a fatal MSI. Of the 5733 cases, 92.9% (*n* = 5326) were recorded on race day, 4.2% (*n* = 239) were euthanised the day after racing, 1.7% (*n* = 97) were euthanised 2 days after racing and 1.2% (*n* = 71) were euthanised on the third day after racing. The overall case incidence rate was 1.49 fatalities per 1000 race starts—a proportion of 2.3% of the individual horses in the study cohort.

A total of 97 potential risk factors were available at the univariable stage of modelling, after which 76 were included as candidates for the final multivariable model. The univariable logistic regression model results are shown in Table S2.

The final model (shown in Table 2) identified statistically significant associations between fatal MSI and 20 risk factors which were retained in the final model. Compared with race starts made on synthetic surface types, starts made on turf or dirt surfaces were at increased odds of fatal MSI. Surfaces recorded as ‘off dirt’—that is, dirt surface types for which the going (or condition) was recorded as anything other than ‘fast’—were associated with the largest increase in odds of fatal MSI (odds ratio [OR] 1.57 [95% confidence interval 1.39–1.77] compared with OR 1.53 [1.37–1.70] for fast dirt and OR 1.40 [1.23–1.58] for turf).

**TABLE 2:** Multivariable logistic regression model results showing risk factors with statistically significant associations with fatal musculoskeletal injury (MSI).

Risk factor	Starts	Fatal MSIs	Per 1000 starts	Odds ratio	95% confidence interval	*p*‐value
Surface						
Synthetic*	412,313	385	0.93	1	—	—
Dirt	2,287,344	3716	1.62	1.53	1.37–1.70	<0.001
Off Dirt^a^	491,726	821	1.67	1.57	1.39–1.77	<0.001
Turf	660,276	811	1.23	1.40	1.23–1.58	<0.001
Field size	Min = 1	Max = 20				
Per additional horse	Median = 8	IQR = 7–10		1.02	1.01–1.04	0.003
Race distance						
Over 6 furlongs*	1,946,916	2699	1.39	1	—	—
Up to 6 furlongs	1,904,743	3034	1.59	1.06	1–1.12	0.05
Purse						
$0–$20,000*	2,119,741	3492	1.65	1	—	—
$20,001–$100,000	1,633,934	2127	1.30	1.14	1.06–1.24	<0.001
$100,001–$16,300,000	97,984	114	1.16	1.37	1.12–1.69	0.003
Void Claim Rule (VCR) in place at track						
No VCR*	2,202,672	3538	1.61	1	—	—
Type 1 (fatality only)	356,635	518	1.45	1	0.91–1.10	0.9
Type 2 (fatality and vet list)	199,317	321	1.61	1.01	0.90–1.13	0.9
Type 3 (fatality, vet list and bled)	279,446	351	1.26	0.85	0.76–0.95	0.005
Type 4 (fatality and van off)	529,969	694	1.31	0.82	0.75–0.89	<0.001
Type 5 (Horseracing Integrity and Safety Authority [HISA] rule)—fatality, vet list, bled and van off	283,620	311	1.10	0.76	0.67–0.85	<0.001
Horse racing age	Min = 2	Max = 13				
Per additional year	Median = 4	IQR = 3–5		1.15	1.11–1.19	<0.001
Horse age at first start						
2 years*	2,192,051	2852	1.30	1	—	—
3+ years	1,659,608	2881	1.74	1.14	1.07–1.21	<0.001
Horse sex						
Female*	1,716,843	2308	1.34	1	—	—
Gelding	1,657,261	2533	1.53	1.12	1.05–1.18	<0.001
Stallion	477,555	892	1.87	1.56	1.44–1.69	<0.001
Horse starts in previous 0–30 days	Min = 0	Max = 7				
Per additional start	Median = 1	IQR = 0–1		0.79	0.75–0.83	<0.001
Horse starts in previous 31–60 days	Min = 0	Max = 5				
Per additional start	Median = 1	IQR = 0–1		1.06	1.02–1.10	0.004
Horse starts in previous 61–90 days	Min = 0	Max = 5				
Per additional start	Median = 1	IQR = 0–1		1.13	1.09–1.17	<0.001
Horse starts in previous 91–180 days	Min = 0	Max = 12				
Per additional start	Median = 1	IQR = 0–3		1.13	1.11–1.16	<0.001
Horse number of starts since trainer change						
Never changed trainer*	1,779,520	2434	1.37	1	—	—
0–1	617,041	1118	1.81	1.18	1.09–1.28	<0.001
2–4	568,393	988	1.74	1.16	1.07–1.26	<0.001
5–8	400,120	614	1.53	1.01	0.92–1.11	0.8
9–105	486,585	579	1.19	0.87	0.78–0.97	0.01
Days since last workout						
0–7 days*	1,069,249	1166	1.09	1	—	—
8–18 days	941,951	1244	1.32	1.17	1.08–1.27	<0.001
19–48 days	953,224	1556	1.63	1.67	1.53–1.81	<0.001
49+ days	887,235	1767	1.99	1.94	1.77–2.12	<0.001
Total distance raced during horse's career						
0–5000 m*	782,925	1098	1.40	1	—	—
5001–15,000 m	1,180,338	2009	1.70	0.75	0.68–0.82	<0.001
15,001–30,000 m	979,444	1456	1.49	0.54	0.48–0.61	<0.001
30,001–200,000 m	908,952	1170	1.29	0.43	0.37–0.51	<0.001
Horse career places	Min = 0	Max = 57				
Per additional place	Median = 4	IQR = 1–8		0.97	0.96–0.98	<0.001
Decimal odds						
8.1+*	2,036,212	2696	1.32	1	—	—
4.1–8.0	965,693	1495	1.55	1.24	1.16–1.32	<0.001
1.0–4.0	849,754	1542	1.81	1.45	1.36–1.55	<0.001
Days since last removed from vet list						
Never been on vet list*	2,678,580	3616	1.35	1	—	—
0–30 days	123,546	284	2.30	1.55	1.37–1.75	<0.001
31–90 days	166,819	345	2.07	1.43	1.27–1.59	<0.001
91–180 days	172,651	395	2.29	1.51	1.36–1.68	<0.001
181–365 days	261,723	480	1.83	1.31	1.19–1.44	<0.001
366–3697 days	448,340	613	1.37	1.11	1.01–1.22	0.03
Horse ever had triage score recorded						
No*	3,771,115	5525	1.47	1	—	—
Yes	80,544	208	2.58	1.55	1.35–1.79	<0.001
Horse claim price						
Not a claimer*	1,388,143	1725	1.24	1	—	—
$1–$5000	859,751	1594	1.85	1.31	1.19–1.45	<0.001
$5001–$10,000	647,404	1122	1.73	1.28	1.16–1.42	<0.001
$10,001–$200,000	956,361	1292	1.35	1.11	1.02–1.2	0.02
Claim price change since previous race						
Decrease of $5000 or more	581,260	943	1.62	1.10	1.02–1.19	0.01
Decrease of <$5000	314,204	607	1.93	1.15	1.04–1.26	0.004
No change*	2,197,842	3087	1.40	1	—	—
Increase (any amount)	758,353	1096	1.45	0.96	0.89–1.04	0.3

*Note*: For categorical variables, a ‘*’ indicates the reference category. For continuous variables, the median, interquartile range (IQR), minimum and maximum are shown in place of the numbers of starts and fatalities.

^a^ ‘Off dirt’ refers to any start made on a Dirt surface, for which track condition was anything other than ‘Fast’.

Race starts made in larger field sizes—number of starters—were at increased odds of fatal MSI. Starts made at or above the third quartile of field size (10 horses) were at odds ratio 1.06 (1.03–1.12) compared with starts made at or below the first quartile (7 horses).

Starts made over shorter race distances were associated with increased odds of fatal MSI. Race starts made at distances less than or equal to the median were at odds ratio 1.06 (1.00–1.12) compared with starts made at distances longer than 6 furlongs.

Races with higher purse sizes were associated with increased odds of fatal MSI. Starts made in races with purse sizes >$100,000 (US Dollars) were 1.37 (1.12–1.69) times more likely to result in a fatal MSI, compared with starts in races with purses ≤$20,000.

**FIGURE 2:**
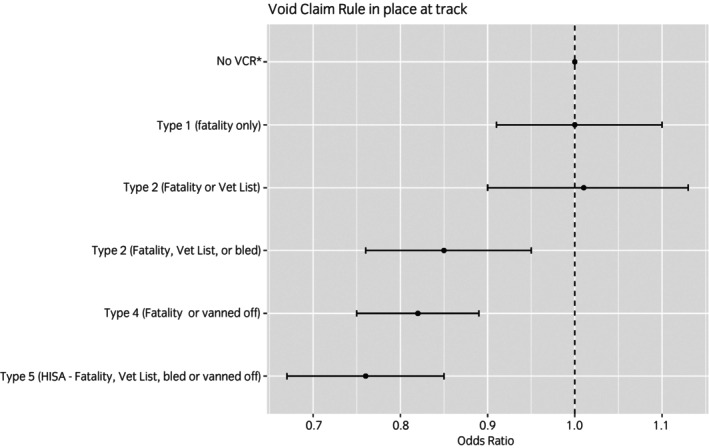
Results from the multivariable logistic regression model, showing the odds ratios (including 95% confidence intervals) for fatal musculoskeletal injury (MSI) associated with each type of Void Claim Rule (VCR). Detailed definitions of the types of VCR are shown in Table 1.

Three types of void claim rule were associated with reduced odds of fatal MSI, compared with starts made in races with no void claim rule in place. The strictest void claim rule—Type 5 (HISA)—was associated with the lowest odds ratio of 0.76 (0.67–0.85) compared with starts made under no void claim rule. Figure 2 shows the results from the final multivariable model for the void claim rule variable.

Older horses were associated with increased odds of fatal MSI. Horses at or above the third quartile of racing age (5 years) were 1.32 (1.23–1.42) times more likely to experience a fatal MSI compared with horses at or below the first quartile (3 years). Horses who made their first race start at racing age of 3 years or older were 1.14 (1.07–1.21) times more likely to experience a fatal MSI at any time during their career, compared with horses whose first race start was made at racing age of 2.

Compared with female horses, both intact and gelded male horses were at increased odds of fatal MSI, with intact males at the greatest increased odds (1.56 [1.44–1.69]).

Over 60% (*n* = 2,338,551) of race starts were made within 30 days of a previous race start. Each additional race start in the previous 0–30 days was associated with a reduced odds of fatal MSI. Each additional race start in the previous 31–60, 61–90 and 91–180 days was associated with increased odds of fatal MSI.

A monotonic relationship was evident in the direction of association between the risk of fatal MSI and time since the horse's previous recorded training workout: compared with race starts made within 7 days of a workout, each additional week between the previous training workout and racing was associated with a further increase in the odds of fatal MSI (OR 1.17 [1.08–1.27] for 8–18 days, increasing to OR 1.94 [1.77–2.12] for 49 days or more).

Changing trainer was associated with an initial increase in odds of fatal MSI for an individual racehorse (OR 1.18 [1.09–1.28] in their first two race starts with a new trainer), with the risk returning to baseline level (i.e., not statistically significantly different to those horses that had not changed trainer) after 5–8 race starts.

Horses with longer total career distances raced were at reduced odds of fatal MSI. Horses with more total place results in their careers were at reduced odds of fatal MSI—those at or above the third quartile of career places (8 places) were 0.81 (0.75–0.87) times less likely to experience a fatal MSI compared with those horses at or below the first quartile (1 career place).

Horses expected to be more competitive according to their betting odds were at increased odds of fatal MSI. Compared with outsiders with decimal odds >8.0, horses with odds 4.1–8.0 and 1.0–4.0 were at progressively increased odds of fatal MSI (1.24 [1.16–1.32] and 1.45 [1.36–1.55], respectively). Note that decimal odds express the multiple of a betting stake that would be paid out, including the stake itself. The favourite to win a race would therefore have the smallest decimal odds of all starters in that race.

Horses who had previously, at any point in their career, had a triage score recorded on an injury reporting form following a race were at increased odds of fatal MSI (1.55 [1.35–1.79]).

Compared with those who had never been on the vet list, horses who had ever been added to and subsequently removed from the vet list were at increased odds of fatal MSI for the rest of their career. The odds of fatality were highest close to when the horses were removed from the vet list and decreased gradually over time but never returned to baseline levels (i.e., equivalent to horses that had never been on the vet list).

Compared with horses that were not raced as claimers, horses racing with any claim price were at increased odds of fatal MSI. Claimers with a price of up to $10,000 were at higher odds of fatal MSI compared with claimers with a price of over $10,000. Horses whose claim price decreased compared with their previous race were at increased odds of fatal MSI compared with horses whose claim price increased or was unchanged (‘unchanged’ includes those horses not racing as claimers).

When validating and refining the final model, horse career length (years) and horse career distance raced were found to be confounded. Horse career distance raced was retained following validation as the overall model fit improved when horse career length (years) was removed. Also, during validation, confounding was identified between the variable ‘days since previous race start’ and each of the four ‘horse starts in previous X–Y days’ (0–30 days, etc.). Following validation, ‘days since previous race start’ was eliminated from the model and the other four (non‐overlapping) variables were retained, which provided the best overall model fit.

No second‐order interaction terms were found to have a statistically significant association with fatal MSI. Mixed‐effects models with horse, trainer and track as random effects found that each of those variables contributed <5% of total variance measured by *R* ^2^. Compared with the single‐level fixed‐effects model, no fixed effects model estimates were altered by more than 10%, and the statistical significance of each risk factor was unchanged in each of the mixed‐effects models. No evidence of a lack of fit was found with the Hosmer–Lemeshow test with 10 degrees of freedom (*p* = 0.4).

## 4 DISCUSSION

Twenty risk factors at the level of the race, horse and track were found to be statistically significantly associated with the risk of fatal MSI in Thoroughbred racehorses in the United States and Canada.

Georgopoulos and Parkin (2016) (henceforth: G + P) is the most directly comparable study to this one, and 10 of the risk factors identified here were also reported in G + P in the same or similar form, with the same direction of effect.^12^ Surface, race distance, purse size, age at first start and horse sex were present in both studies in the same form. In G + P, two variables ‘time with same trainer’ and ‘number of days between race starts since change of trainer’ are encapsulated in the variable ‘horse number of starts since changing trainer’ which was retained in the final model of the present study. The direction of effect—that risk of fatal MSI reduces with time spent with the same trainer—was the same, although the form used here allowed the identification of an initial increase in risk immediately after a horse changes trainer.

Track surface and track condition have been the subject of intense discussion in recent years, especially with respect to their potential association with fatal MSI. In this study, various forms of both variables were assessed, and eventually, the variable that was retained in the final model was a combination of the two with four levels: Synthetic, Turf, Dirt and Off Dirt. Of the various track conditions that were recorded in the EID, only Off Dirt (i.e., Dirt tracks with any condition other than Fast) was found to be associated with a different level of risk of fatal MSI compared with starts on the reference track condition for the relevant surface. This emphasises the multifactorial nature of the risk of fatal MSI—undue focus on any single variable such as surface or track condition is likely to be unhelpful for the broader goal of reducing the incidence of fatal MSI.

G + P reported that the odds of fatal MSI reduced the lower a horse finished in the field in their previous race, and the higher their odds rank in the previous race. In other words, the more competitive a horse was in their previous race, the higher their risk of fatal MSI. The closest corresponding risk factor in the present study is ‘decimal odds’, which found that horses expected to be competitive in the current race were at increased odds of fatal MSI. The present study also identified that horses with more previous place results (i.e., a competitive finish) in their career were at reduced odds of fatal MSI. This may in part be a proxy measure for career length, hence the association could be a ‘healthy horse effect’, that is, healthier horses are able to keep racing and potentially achieving more place results and it is the fact that they are ‘healthy’ which is the true causal factor and not the number of places itself.

In G + P it was found that horses who had previously been on the vet list were at increased odds of fatal MSI. They also reported that those horses that had previously been scratched (a scratch is when they are ruled unsuitable to race during the pre‐race veterinary exam) or recorded as injured at some point in their career were at increased odds of fatal MSI. Here, the corresponding risk factors were ‘days since last removed from the vet list’ and ‘ever had a triage score recorded’—the more complete form of the vet list data that was available for the present study allowed different forms of these variables to be assessed compared with those that were available for G + P. It is important to note that in the form of the variable used here, each horse must have passed a vet exam before they were removed from the vet list: and yet, they remain at increased risk of fatal MSI for a very long period of time afterwards. Although the odds ratio reduced with increased time since coming off the vet list, it never returned to baseline. There are several reasons a horse would be added to the vet list, but this result suggests that on average, a horse exhibiting clinical signs such that they are put on the vet list may have sustained some form of pathology that puts them at increased risk of fatal MSI, sometimes months or potentially years later, even in the apparent absence of the original clinical signs.

The presence of pathology which may cause increased risk of fatal MSI is also demonstrated by the result that if a horse had ever previously had a triage score recorded, it was at increased risk of fatal MSI for the rest of its racing career. There is some overlap between this variable and the vet list variable in the final model, since a triage score would usually be accompanied by the horse being added to the vet list, but these variables were not found to be significantly confounded and therefore both were retained in the final model.

The wide range of reasons for being on the vet list or having previously received a triage score means that these risk factors are somewhat imprecise in their ‘veterinary diagnoses’. It is reasonable to assume that amongst the several reasons for being classified as exposed to either variable, some are more strongly associated with fatal MSI and indeed some are not associated at all. It would therefore be of significant benefit to future (predictive) models if greater clarity on the underlying veterinary diagnoses or pathologies associated with these variables could be made available in future EID reporting. This, alongside an ability to interrogate full veterinary histories of the whole racing population, would likely advance the future predictive, and therefore preventative, ability of work in this area.

Results related to claiming rules are also a potential proxy for underlying pathology (known or unknown to the owner, unknown to the data set) since these variables are effectively related to choices made by horse owners given the information available to them at the time. It is the owner's choice to enter the horse into a claiming race; it is their choice to change a horse's claim price in subsequent races. A decreasing claim price may indicate an owner who is keen to sell their horse—a large decrease in claim price may suggest this is a more urgent desire, which could be motivated by many potential factors. The association between the presence of void claim rules at the track and a reduced likelihood of fatal MSI adds further context to claim price as a proxy measure. It is perhaps not unreasonable to assume that one reason for the association with the void claim rules is that the rule specifically deters owners from entering horses with known pathology into races. The results here—in particular, the result that the Type 5 (HISA) rule was most ‘protective’ against fatality—support that reasoning and suggest that the presence of a void claim rule influences the choices made by owners as intended, with the ultimate outcome being a reduction in the risk of (fatal) injury.

Both variables ‘number of races in [time period]’ and ‘days since last workout’ combine to a result that indicates recent exercise is associated with a reduced odd of fatal MSI, but too much exercise over a sustained period can also increase the risk. This is consistent with the results of previous studies showing that exercise‐driven bone adaptation is a key factor in racehorse injury.^33^, ^51^, ^52^, ^53^ These results are in agreement that a suitable exercise routine—covering both training and racing—is an essential aspect of managing risk to racehorses. It is also important to recognise that ‘suitable exercise routines’ will be different for each horse and that it would be unreasonable to expect any such studies to identify the ‘optimal routine’ that would suit the needs of all horses.

Compared with G + P, the new risk factors and additional insight into the EID provided by 10 years' of extra data, plus new data sources relating to training, veterinary inspections and sales, demonstrate the value of treating risk management in equine sports as an ongoing process, and not as a one‐off event. The increased breadth (more race starts recorded) and depth (new data sources) combined to provide a more detailed model than was previously available. Ensuring that all stakeholders are aware of these risk factors is a critical step in the sustained efforts to further reduce risk and maximise equine welfare in racing.

The most significant limitations to the present study are related to the lack of availability of data in some domains which are known to contain important potential risk factors. The EID is comprehensive in terms of racing records and racing injury records. However, detailed records from training are not routinely collected, other than the date of the horse's previous workout before each race. A more comprehensive record of training beyond the most recent workout before each race was available for some horses but was incomplete for the full EID, and therefore it was not possible to include it in this study. Records of veterinary management are not collected in the EID, other than proxy measurements such as the vet list or triage score, and only when related to a race start. The presence of these variables in the final model here suggests that both domains—training and veterinary history—may contain other important risk factors. Adding more comprehensive versions of both data sets into future analyses may potentially lead to new insights into risk factors that are currently unavailable for study.

Notable potential risk factors that were not present in the final model include those related to sales before their racing careers began and those related to race‐day medication. Preliminary analysis of sales‐related variables in a separate, in‐progress study has identified previously unknown associations between sales status at an early age and eventual career length. Compared with horses who were not sold, horses who were sold at age 1 or 2 years had, on average, racing careers that began when they were younger, included more race starts, and also ended when they were younger. The use of race‐day phenylbutazone was found to be associated with increased odds of fatality in South American racing, and race‐day furosemide was associated with increased odds of sudden death in North America^28^, ^39^—both medications were associated with increased odds of fatal MSI at the univariable stage of modelling in the current study, but neither was statistically significant in the final multivariable model.

## 5 CONCLUSIONS

Twenty risk factors at the level of the horse, trainer, race and track were found to be associated with race‐related death or euthanasia due to fatal MSI. Some of the risk factors identified here have been present in previous studies; others were available for the first time in this study. This highlights the importance of treating risk management as an ongoing process—new data sources added to a growing database have provided new insights into the risk factors affecting racing Thoroughbreds.

These results allow a refinement of possible risk profiles for individual horses, potentially enabling those horses who are at greatest risk of fatal MSI to be identified in advance. In particular, racing regulatory veterinarians could undertake additional pre‐race examinations of horses who have ever previously had a triage score recorded, horses who have ever previously been added to the vet list, and horses that have recently changed trainers.

These results validate the effectiveness of void claim rules and motivate further investigation into the exact mechanism that leads to horses racing as claimers being at increased odds of fatal MSI. Horses racing as claimers could also be prioritised for pre‐race vet examination, especially if their claim price has decreased from their previous race start.

## FUNDING INFORMATION

The study was supported by ‘The Jockey Club’.

## CONFLICT OF INTEREST STATEMENT

The authors have declared no conflicting interests.

## AUTHOR CONTRIBUTIONS

**Euan David Bennet:** Conceptualization; methodology; software; formal analysis; validation; investigation; funding acquisition; writing – original draft; writing – review and editing; visualization; project administration; data curation. **Tim D. H. Parkin:** Conceptualization; methodology; validation; supervision; funding acquisition; writing – review and editing; project administration; investigation.

## DATA INTEGRITY STATEMENT

E. Bennet had full access to all the data in the study and is responsible for data integrity and accuracy of the analysis.

## ETHICAL ANIMAL RESEARCH

Research ethics committee oversight not currently required by this journal: data provided by a sports regulator were analysed.

## INFORMED CONSENT

Explicit owner‐informed consent for inclusion of animals in this study was not stated. Representatives of the Jockey Club gave consent for use of their data.

## ANTIMICROBIAL STEWARDSHIP POLICY

Not applicable.

## Supporting information


**Table S1.** All potential risk factors available for investigation, along with their categorisations and a description where relevant.

**Table S2.** Univariable model results for the 76 risk factors for fatal musculoskeletal injury shown in Table S1.

## Data Availability

The data that support the findings of this study may be available from The Jockey Club. Restrictions apply to the availability of the complete data, which were used under licence for this study.
