# Supplementary material for: Novel risk factors associated with fatal musculoskeletal injury in Thoroughbreds in North American racing (2009–2023)
Source: Equine Vet J. 2025 Mar 25;58(1):20–30. doi: 10.1111/evj.14503 (PMC12699123; doi:10.1111/evj.14503)
Supplement: Supplementary file 1 — Table S1. All potential risk factors available for investigation, along with their categorisations and a description where relevant. [file EVJ-58-20-s002.pdf]

Table S1: Table shows all potential risk factors available for investigation, along with their categorisations, and a description where relevant.

**Potential risk factor**	**Categorisation**	**Description**
Country	Binary	Either USA or Canada.
Post position	Continuous	Position of horse by number at the start of the race.
Purse size	Categorical	Quartiles rounded to the nearest $10,000 (US Dollars), then lowest 2 quartiles combined.
Surface	Categorical	Either Dirt, Turf, or Synthetic (all-weather).
Course type	Categorical	A more detailed version of the 'Surface' variable.
Track condition	Binary	Classified as 'Fast/Firm' (for Dirt and Turf surfaces respectively), or 'Other'.
Horse claim price	Categorical	Claim price in US Dollars.
Race moved off turf	Binary	Indicator whether the race was originally scheduled to take place on a Turf track, then moved due to poor weather conditions.
Weight carried	Continuous	Weight in kg carried by the horse.
Sex restriction	Categorical	Indicator whether the race was restricted to certain sexes of horse.
Last start surface	Categorical	The track surface for the horse's previous race start.
Days since last workout	Categorical	Categorised by quartiles.
Last workout surface	Categorical	The track surface for the horse's previous workout.
Field size	Continuous	The number of horses that started the race.
Favourite indicator	Binary	Indicator whether the horse was the favourite to win the race.
Track sealed indicator	Binary	Indicator whether the race track was 'sealed' due to poor weather conditions. Only applies to Dirt tracks.
Horse on lasix this race	Binary	Indicator whether the horse was recorded as having been given furosemide (lasix) on this race day.
Horse on bute this race	Binary	Indicator whether the horse was recorded as having been given phenylbutazone (bute) on this race day.
Horse on ABM this race	Binary	Indicator whether the horse was recorded as having been given adjunct bleeder medication (ABM) on this race day.
Days since last race	Continuous	Number of days since the horse's previous race start.
Layoff period	Categorical	Days since last race in categorical form. Categorised into 30-day periods.

Potential risk factor	Categorisation	Description
HISA status	Categorical	The status of the racetrack with respect to the jurisdiction of the Horseracing Integrity and Safety Authority (HISA).
Horse sex	Categorical	Classified as 'female', 'gelding', or 'stallion'.
Horse racing age	Continuous	
Horse age at first race start	Categorical	Grouped into 'age 2' and 'age 3 or older'.
Race distance (furlongs)	Categorical	Grouped into 'up to 6 furlongs' or 'over 6 furlongs'.
Purse change	Categorical	Change in purse size from the horse's previous race.
Race type	Categorical	Classification of the type of race.
Decimal odds	Categorical	Categorised by quartiles rounded to whole numbers.
Speed this race (km/h)	Continuous	
High speed (>40 km/h)	Binary	Indicator whether the horse was raced at faster than 40 km/h.
Horse career length (starts)	Categorical	Categorised by quartiles.
Horse career length (days)	Categorical	Categorised by quartiles.
Horse career race distance (m)	Categorical	Categorised by quartiles rounded to the nearest 5000m
Horse career distance at high speed (m)	Categorical	Categorised by quartiles.
Horse career length (years)	Categorical	Categorised into 0, 1, 2, and 3+ years.
Number of starts in previous 0-30 days	Continuous	
Number of starts in previous 0-60 days	Continuous	
Number of starts in previous 0-90 days	Continuous	
Number of starts in previous 0-180 days	Continuous	
Number of starts in previous 30-60 days	Continuous	
Number of starts in previous 60-90 days	Continuous	
Number of starts in previous 90-180 days	Continuous	
Career wins	Continuous	

Potential risk factor	Categorisation	Description
Career places	Continuous	
Starts since changing trainer	Categorical	Categorised into never changed trainer, and quartiles for those that did change trainer.
Speed in previous start (km/h)	Categorical	Categorised into 0-40.0, 40.1-50.0, 50.1-75.0
Distance of previous race (m)	Categorical	Categorised into quartiles.
Change in speed from previous race	Categorical	
Change in claim price from previous race	Categorical	
Horse started this race as a claimer	Binary	
Horse started their previous race as a claimer	Binary	
Horse has ever previously been a claimer	Binary	
Number of previous starts as a claimer	Categorical	Categorised into quartiles.
Number of previous starts in state-bred races	Continuous	
Number of previous injuries	Categorical	
Horse has ever previously been injured	Binary	
Horse was triaged in their previous start	Binary	
Horse has ever previously been triaged	Binary	
Horse was added to the vetlist at this race	Binary	
Horse has ever previously been on the vetlist	Binary	
Number of times horse has previously been on the vetlist	Categorical	Categorised into 0, 1, or 2+ times.
Days since horse was last removed from the vetlist	Categorical	Categorised into periods corresponding to 1, 3, 6, and 12+ months.

Potential risk factor	Categorisation	Description
Horse was returning from 60-day layoff	Binary	
Number of career 60-day layoffs	Categorical	Categorised into 0, 1, 2, or 3+.
Horse has ever previously been in 60-day layoff	Binary	
Number of starts since last 60-day layoff	Categorical	Categorised into quintiles for those that had previously been in layoff.
Number of days since last 60-day layoff	Categorical	Categorised into 0-30, 31-90, and 91+ days.
Horse's last start was on a sealed dirt track	Binary	
Horse has ever raced on a sealed dirt track	Binary	
Number of starts since horse last raced on a sealed dirt track	Categorical	Categorised into quintiles for those that had previously raced on a sealed track.
Number of days since horse last raced on a sealed dirt track	Categorical	Categorised into 0-60, 61-180, and 181+ days.
Horse raced on a sealed dirt track in last 30 days	Binary	
Horse raced on a sealed dirt track in last 60 days	Binary	
Horse raced on a sealed dirt track in last 180 days	Binary	
Void claim rule in place at track	Categorical	Either no rule was in place, or one of five different types was used.
