# Supplementary material for: Novel risk factors associated with fatal musculoskeletal injury in Thoroughbreds in North American racing (2009–2023)
Source: Equine Vet J. 2025 Mar 25;58(1):20–30. doi: 10.1111/evj.14503 (PMC12699123; doi:10.1111/evj.14503)
Supplement: Supplementary file 2 — Table S2. Univariable model results for the 76 risk factors for fatal musculoskeletal injury shown in Table S1. [file EVJ-58-20-s001.pdf]

Equine Veterinary Journal BEVA

Table S2: Univariable model results for the 76 risk factors for fatal musculoskeletal injury (MSI) shown in Table S1. For categorical risk factors, a * indicates the reference category. For continuous risk factors, the median, interquartile range (IQR), minimum and maximum values are shown instead of the numbers of starts and fatalities.

**Potential risk factor**	**Starts**	**Fatal MSIs**	**Odds ratio**	**95% confidence interval**	**p-value**
Country					
Canada*	302864	315 (0.1%)	1	-	-
USA	3548795	5418 (0.2%)	1.47	1.31-1.65	<0.001
Post position	min = 1	max = 20			
Per additional position number	median = 4	IQR = 2-6	1.01	1-1.02	0.06
Purse (US Dollars)					
$0-$20,000*	2119741	3492 (0.2%)	1	-	-
$20,001-$100,000	1633934	2127 (0.1%)	0.79	0.75-0.83	<0.001
$100,001-$16,300,000	97984	114 (0.1%)	0.71	0.58-0.85	<0.001
Surface					
Dirt*	2779070	4537 (0.2%)	1	-	-
Synth	412313	385 (0.1%)	0.57	0.51-0.63	<0.001
Turf	660276	811 (0.1%)	0.75	0.7-0.81	<0.001

Potential risk factor	Starts	Fatal MSIs	Odds ratio	95% confidence interval	p-value
Course type					
All Weather Track*	412313	385 (0.1%)	1	-	-
Dirt	2751447	4476 (0.2%)	1.74	1.57-1.94	<0.001
Downhill turf	10905	26 (0.2%)	2.56	1.68-3.73	<0.001
Inner track	27623	61 (0.2%)	2.37	1.79-3.08	<0.001
Inner turf	39220	40 (0.1%)	1.09	0.78-1.49	0.6
Outer turf	9064	11 (0.1%)	1.3	0.67-2.25	0.4
Turf	601087	734 (0.1%)	1.31	1.16-1.48	<0.001
Track condition					
Fast/Firm*	3253062	4803 (0.1%)	1	-	-
Other	598597	930 (0.2%)	1.05	0.98-1.13	0.2
Horse claim price (US Dollars)					
not a claimer*	1388143	1725 (0.1%)	1	-	-
$1-$5,000	859751	1594 (0.2%)	1.49	1.39-1.6	<0.001
$5,001-$10,000	647404	1122 (0.2%)	1.4	1.29-1.5	<0.001
$10,001-$200,000	956361	1292 (0.1%)	1.09	1.01-1.17	0.02
Race moved off turf					

**Potential risk factor**	**Starts**	**Fatal MSIs**	**Odds ratio**	**95% confidence interval**	**p-value**
No*	3732404	5596 (0.1%)	1	-	-
Yes	119255	137 (0.1%)	0.77	0.64-0.9	0.002
Weight carried	min = 93	max = 154			
Per additional kg	median = 120	IQR = 118-122	0.99	0.98-1	0.04
Sex restriction					
Open*	2153261	3445 (0.2%)	1	-	-
C & G (colts and geldings)	9106	17 (0.2%)	1.17	0.7-1.82	0.5
F & M (fillies and mares)	1343178	1870 (0.1%)	0.87	0.82-0.92	<0.001
F (fillies)	346095	401 (0.1%)	0.72	0.65-0.8	<0.001
M (mares only)	19	0 (0%)	0.01	NA-0.48	0.9
Last start surface					
Dirt*	2599301	4217 (0.2%)	1	-	-
first start	240634	251 (0.1%)	0.64	0.56-0.73	<0.001
Synth	386463	429 (0.1%)	0.68	0.62-0.75	<0.001
Turf	625261	836 (0.1%)	0.82	0.76-0.89	<0.001
Days since last workout					

Potential risk factor	Starts	Fatal MSIs	Odds ratio	95% confidence interval	p-value
0-7*	1069249	1166 (0.1%)	1	-	-
8-18	941951	1244 (0.1%)	1.21	1.12-1.31	<0.001
19-48	953224	1556 (0.2%)	1.5	1.39-1.62	<0.001
49+	887235	1767 (0.2%)	1.83	1.7-1.97	<0.001
Last workout surface					
Dirt*	3342896	5148 (0.2%)	1	-	-
first start	1880	2 (0.1%)	0.69	0.11-2.13	0.6
Synth	466282	538 (0.1%)	0.75	0.68-0.82	<0.001
Turf	40601	45 (0.1%)	0.72	0.53-0.95	0.03
Field size	min = 1	max = 20			
Per additional starter	median = 8	IQR = 8-10	1.01	1-1.02	0.2
Favourite in race					
No*	3338933	4802 (0.1%)	1	-	-
Yes	512726	931 (0.2%)	1.26	1.18-1.35	<0.001
Track sealed					
No*	3552024	5247 (0.1%)	1	-	-
Yes	299635	486 (0.2%)	1.1	1-1.2	0.05

Potential risk factor	Starts	Fatal MSIs	Odds ratio	95% confidence interval	p-value
Horse on Lasix this race					
No*	266693	313 (0.1%)	1	-	-
Yes	3584966	5420 (0.2%)	1.29	1.15-1.45	<0.001
Horse on bute this race					
No*	3604781	5331 (0.1%)	1	-	-
Yes	246878	402 (0.2%)	1.1	0.99-1.22	0.06
Horse on ABM this race					
No*	3749781	5489 (0.1%)	1	-	-
Yes	101878	244 (0.2%)	1.64	1.44-1.86	<0.001
Days since last race	min = 1	max = 2633			
Per additional day	median = 24	IQR = 16-39	1	01-Jan	0.001
Layoff period (days)					
0-30*	2338386	3485 (0.1%)	1	-	-
31-60	795328	1417 (0.2%)	1.2	1.12-1.27	<0.001
61-90	163405	269 (0.2%)	1.1	0.97-1.25	0.1
91-120	66199	98 (0.1%)	0.99	0.81-1.21	0.9

Potential risk factor	Starts	Fatal MSIs	Odds ratio	95% confidence interval	p-value
>120	243411	202 (0.1%)	0.56	0.48-0.64	<0.001
first start	244930	262 (0.1%)	0.72	0.63-0.81	<0.001
Horseracing Integrity and Safety Authority (HISA) status					
YES*	2658273	3883 (0.1%)	1	-	-
NO	699915	1234 (0.2%)	1.21	1.13-1.29	<0.001
NO SIMULCAST	42176	70 (0.2%)	1.14	0.89-1.43	0.3
CANADA	270926	284 (0.1%)	0.72	0.63-0.81	<0.001
NOT APPLICABLE	180369	262 (0.1%)	0.99	0.88-1.12	0.9
Horse sex					
female	1716843	2308 (0.1%)	1	-	-
gelding	1657261	2533 (0.2%)	1.14	1.07-1.2	<0.001
stallion	477555	892 (0.2%)	1.39	1.29-1.5	<0.001
Horse racing age	min = 2	max = 13			
Per additional year	median = 4	IQR = 3-5	1.01	1-1.03	0.1
Horse age at first race start (years)					
2*	2192051	2852 (0.1%)	1	-	-

**Potential risk factor**	**Starts**	**Fatal MSIs**	**Odds ratio**	**95% confidence interval**	**p-value**
3+	1659608	2881 (0.2%)	1.33	1.27-1.41	<0.001
Race distance (furlongs)	min = 0.7	max = 18			
Per additional furlong	median = 6.5	IQR = 6-8	0.94	0.92-0.96	<0.001
Purse change from previous race					
No change*	1305321	1901 (0.1%)	1	-	-
Decrease	1344258	2145 (0.2%)	1.1	1.03-1.17	0.004
Increase	1202080	1687 (0.1%)	0.96	0.9-1.03	0.3
Race type					
Allowance	510217	620 (0.1%)	1	-	-
Claimer	1856533	3032 (0.2%)	1.34	1.23-1.47	<0.001
Maiden Claimer	786165	1199 (0.2%)	1.26	1.14-1.38	<0.001
Other	698744	882 (0.1%)	1.04	0.94-1.15	0.5
Decimal odds					
8.1-1000.0*	2036212	2696 (0.1%)	1	-	-
4.1-8.0	965693	1495 (0.2%)	1.17	1.1-1.25	<0.001
1.0-4.0	849754	1542 (0.2%)	1.37	1.29-1.46	<0.001

Potential risk factor	Starts	Fatal MSIs	Odds ratio	95% confidence interval	p-value
Speed this race (km per hour)	min = 18.4	max = 74.9			
Per additional km/h	median = 41.4	IQR = 37.8-41.4	0.58	0.58-0.59	<0.001
High speed (speed > 40km/h)					
No*	2185283	4784 (0.2%)	1	-	-
Yes	1664937	948 (0.1%)	0.26	0.24-0.28	<0.001
Data unavailable	1439	1 (0.1%)			
Career length starts					
1-5	1077596	1594 (0.1%)	1	-	-
6-11	904977	1499 (0.2%)	1.12	1.04-1.2	0.002
10-21	917880	1360 (0.1%)	1	0.93-1.08	1
22-129	951206	1280 (0.1%)	0.91	0.84-0.98	0.01
Career length days					
0-157*	964297	1538 (0.2%)	1	-	-
158-428	964287	1538 (0.2%)	1	0.93-1.07	1
429-820	960912	1322 (0.1%)	0.86	0.8-0.93	<0.001
821-4156	962163	1335 (0.1%)	0.87	0.81-0.94	<0.001

Potential risk factor	Starts	Fatal MSIs	Odds ratio	95% confidence interval	p-value
Career race distance (m)					
0-5000*	782925	1098 (0.1%)	1	-	-
5001-15000	1180338	2009 (0.2%)	1.21	1.13-1.31	<0.001
15001-30000	979444	1456 (0.1%)	1.06	0.98-1.15	0.1
30001-200000	908952	1170 (0.1%)	0.92	0.85-1	0.04
Career distance at high speed (m)					
0-1408*	343039	561 (0.2%)	1	-	-
1409-4828	958637	1487 (0.2%)	0.95	0.86-1.05	0.3
4829-13176	956674	1398 (0.1%)	0.89	0.81-0.99	0.02
13177-179839	958007	1158 (0.1%)	0.74	0.67-0.82	<0.001
Data unavailable	635302	1129 (0.2%)			
Career length (years)					
0*	1697819	2764 (0.2%)	1	-	-
1	997574	1365 (0.1%)	0.84	0.79-0.9	<0.001
2	581645	805 (0.1%)	0.85	0.79-0.92	<0.001
3+	574621	799 (0.1%)	0.85	0.79-0.92	<0.001
Horse starts in previous 0-30 days					

**Potential risk factor**	**Starts**	**Fatal MSIs**	**Odds ratio**	**95% confidence interval**	**p-value**
0*	1513273	2248 (0.1%)	1	-	-
1	1925878	2981 (0.2%)	1.04	0.99-1.1	0.1
2-7	412508	504 (0.1%)	0.82	0.75-0.9	<0.001
Horse starts in previous 0-60 days					
0*	717945	831 (0.1%)	1	-	-
1	1211909	1885 (0.2%)	1.34	1.24-1.46	<0.001
2	1208394	1985 (0.2%)	1.42	1.31-1.54	<0.001
3-9	713411	1032 (0.1%)	1.25	1.14-1.37	<0.001
Horse starts in previous 0-90 days					
0*	554540	562 (0.1%)	1	-	-
1	713133	990 (0.1%)	1.37	1.24-1.52	<0.001
2	931231	1509 (0.2%)	1.6	1.45-1.76	<0.001
3	849107	1392 (0.2%)	1.62	1.47-1.79	<0.001
4-12	803648	1280 (0.2%)	1.57	1.42-1.74	<0.001
Horse starts in previous 0-180 days					
0-2*	1292821	1501 (0.1%)	1	-	-
3-4	981547	1492 (0.2%)	1.31	1.22-1.41	<0.001

Potential risk factor	Starts	Fatal MSIs	Odds ratio	95% confidence interval	p-value
5-6	889936	1564 (0.2%)	1.51	1.41-1.63	<0.001
7-21	687355	1176 (0.2%)	1.47	1.37-1.59	<0.001
Horse starts in previous 30-60 days					
0*	1452066	1804 (0.1%)	1	-	-
1	1677542	2773 (0.2%)	1.33	1.25-1.41	<0.001
2-5	722051	1156 (0.2%)	1.29	1.2-1.39	<0.001
Horse starts in previous 60-90 days					
0*	1769110	2213 (0.1%)	1	-	-
1	1434756	2436 (0.2%)	1.36	1.28-1.44	<0.001
2-5	647793	1084 (0.2%)	1.34	1.24-1.44	<0.001
Horse starts in previous 90-180 days					
0*	1447992	1696 (0.1%)	1	-	-
1	531621	761 (0.1%)	1.22	1.12-1.33	<0.001
2-3	1243756	2183 (0.2%)	1.5	1.41-1.6	<0.001
4-12	628290	1093 (0.2%)	1.49	1.38-1.6	<0.001
Career wins	min = 0	max = 44			
Per additional win	median = 1	IQR = 0-3	0.99	0.98-1	0.01

Potential risk factor	Starts	Fatal MSIs	Odds ratio	95% confidence interval	p-value
Career places	min = 0	max = 57			
Per additional placing	median = 4	IQR = 1-8	0.99	0.98-0.99	<0.001
Starts since trainer change					
never changed trainer*	1779520	2434 (0.1%)	1	-	-
0-1	617041	1118 (0.2%)	1.33	1.23-1.42	<0.001
2-4	568393	988 (0.2%)	1.27	1.18-1.37	<0.001
5-8	400120	614 (0.2%)	1.12	1.03-1.23	0.01
9-105	486585	579 (0.1%)	0.87	0.79-0.95	0.003
Speed in previous start (km/h)					
0-40.0	2030033	3208 (0.2%)	1	-	-
40.1-50.0	1229878	1653 (0.1%)	0.85	0.8-0.9	<0.001
50.1-75.0	345427	609 (0.2%)	1.12	1.02-1.22	0.01
first start	246321	263 (0.1%)	0.68	0.59-0.76	<0.001
Distance in previous start (m)					
0-1200	886032	1484 (0.2%)	1	-	-
1201-1300	898382	1398 (0.2%)	0.93	0.86-1	0.05
1301-1600	577987	862 (0.1%)	0.89	0.82-0.97	0.007

Potential risk factor	Starts	Fatal MSIs	Odds ratio	95% confidence interval	p-value
1601-3600	1244328	1727 (0.1%)	0.83	0.77-0.89	<0.001
first start	244930	262 (0.1%)	0.64	0.56-0.73	<0.001
Speed change from previous start					
no change	653637	403 (0.1%)	1	-	-
decrease	1577839	4683 (0.3%)	4.83	4.36-5.35	<0.001
increase	1620183	647 (0%)	0.65	0.57-0.73	<0.001
Claim price change from previous start					
no change	2197842	3087 (0.1%)	1	-	-
decrease >= $5,000	581260	943 (0.2%)	1.16	1.07-1.24	<0.001
decrease < $5,000	314204	607 (0.2%)	1.38	1.26-1.5	<0.001
increase	758353	1096 (0.1%)	1.03	0.96-1.1	0.4
Claimer in this race					
No*	1388143	1725 (0.1%)	1	-	-
Yes	2463516	4008 (0.2%)	1.31	1.24-1.39	<0.001
Claimer in last race					
No*	1566915	2034 (0.1%)	1	-	-
Yes	2284744	3699 (0.2%)	1.25	1.18-1.32	<0.001

**Potential risk factor**	**Starts**	**Fatal MSIs**	**Odds ratio**	**95% confidence interval**	**p-value**
Ever been a claimer					
No*	942707	1153 (0.1%)	1	-	-
Yes	2908952	4580 (0.2%)	1.29	1.21-1.37	<0.001
Number of previous claimer races					
0*	942707	1153 (0.1%)	1	-	-
1	323731	507 (0.2%)	1.28	1.15-1.42	<0.001
2-5	806252	1362 (0.2%)	1.38	1.28-1.49	<0.001
6-13	882131	1456 (0.2%)	1.35	1.25-1.46	<0.001
14-120	896838	1255 (0.1%)	1.14	1.06-1.24	<0.001
Number of previous starts in state-bred races	min = 0	max = 100			
Per additional start	median = 0	IQR = 0-3	0.99	0.99-1	0.008
Career number of previous injuries	min=0	max=7			
Per additional injury	median=0	IQR=0-0	1.29	1.18-1.41	<0.001
Ever previously been injured					
No*	3683838	5393 (0.1%)	1	-	-

**Potential risk factor**	**Starts**	**Fatal MSIs**	**Odds ratio**	**95% confidence interval**	**p-value**
Yes	167821	340 (0.2%)	1.38	1.24-1.54	<0.001
Triage in last start					
No*	3843839	5712 (0.1%)	1	-	-
Yes	7820	21 (0.3%)	1.81	1.14-2.7	0.007
Ever been triaged					
No*	3771115	5525 (0.1%)	1	-	-
Yes	80544	208 (0.3%)	1.76	1.53-2.02	<0.001
On vetlist this race					
No*	3818969	5667 (0.1%)	1	-	-
Yes	32690	66 (0.2%)	1.36	1.06-1.72	0.01
Ever previously been on vetlist					
No*	2645890	3550 (0.1%)	1	-	-
Yes	1205769	2183 (0.2%)	1.35	1.28-1.42	<0.001
Number of times horse has previously been on vetlist					
0*	2645890	3550 (0.1%)	1	-	-

Potential risk factor	Starts	Fatal MSIs	Odds ratio	95% confidence interval	p-value
1	752612	1319 (0.2%)	1.31	1.23-1.39	<0.001
2+	453157	864 (0.2%)	1.42	1.32-1.53	<0.001
Days since last removed from vetlist					
never been on vetlist*	2678580	3616 (0.1%)	1	-	-
0-30	123546	284 (0.2%)	1.7	1.51-1.92	<0.001
31-90	166819	345 (0.2%)	1.53	1.37-1.71	<0.001
91-180	172651	395 (0.2%)	1.7	1.53-1.88	<0.001
181-365	261723	480 (0.2%)	1.36	1.23-1.49	<0.001
366-3697	448340	613 (0.1%)	1.01	0.93-1.1	0.8
Returning from layoff					
No*	3378644	5164 (0.2%)	1	-	-
Yes	473015	569 (0.1%)	0.79	0.72-0.86	<0.001
Number of layoffs in career					
0*	1151723	1930 (0.2%)	1	-	-
1	1045690	1558 (0.1%)	0.89	0.83-0.95	<0.001
2	687570	966 (0.1%)	0.84	0.78-0.91	<0.001
3-19	966676	1279 (0.1%)	0.79	0.74-0.85	<0.001

**Potential risk factor**	**Starts**	**Fatal MSIs**	**Odds ratio**	**95% confidence interval**	**p-value**
Ever been in layoff					
No*	1151723	1930 (0.2%)	1	-	-
Yes	2699936	3803 (0.1%)	0.84	0.8-0.89	<0.001
Starts since last layoff					
never been in layoff*	1151723	1930 (0.2%)	1	-	-
0	473015	569 (0.1%)	0.72	0.65-0.79	<0.001
1	391404	462 (0.1%)	0.7	0.64-0.78	<0.001
2-3	589852	764 (0.1%)	0.77	0.71-0.84	<0.001
4-7	678512	1028 (0.2%)	0.9	0.84-0.97	0.009
8-90	567153	980 (0.2%)	1.03	0.95-1.11	0.4
Days since last layoff					
never been in layoff*	1151723	1930 (0.2%)	1	-	-
0-30	807364	893 (0.1%)	0.66	0.61-0.71	<0.001
31-90	725711	971 (0.1%)	0.8	0.74-0.86	<0.001
91-2003	1166861	1939 (0.2%)	0.99	0.93-1.06	0.8
Track sealed in horse's last start					
No*	3570366	5256 (0.1%)	1	-	-

Potential risk factor	Starts	Fatal MSIs	Odds ratio	95% confidence interval	p-value
Yes	281293	477 (0.2%)	1.15	1.05-1.26	0.003
Ever raced on sealed track					
No*	2096997	3050 (0.1%)	1	-	-
Yes	1754662	2683 (0.2%)	1.05	1-1.11	0.06
Starts since last start on sealed track					
never raced on sealed track*	2096997	3050 (0.1%)	1	-	-
0	281293	477 (0.2%)	1.17	1.06-1.28	0.002
1	229247	372 (0.2%)	1.12	1-1.24	0.05
2-4	479961	723 (0.2%)	1.04	0.95-1.12	0.4
5-8	353558	559 (0.2%)	1.09	0.99-1.19	0.07
9-109	410603	552 (0.1%)	0.92	0.84-1.01	0.09
Days since last start on sealed track					
never raced on sealed track*	2096997	3050 (0.1%)	1	-	-
0-60	529432	879 (0.2%)	1.14	1.06-1.23	<0.001
61-180	465283	808 (0.2%)	1.19	1.1-1.29	<0.001
181-3746	759947	996 (0.1%)	0.9	0.84-0.97	0.004
Horse raced on sealed track in last 30 days					

Potential risk factor	Starts	Fatal MSIs	Odds ratio	95% confidence interval	p-value
No*	3565828	5278 (0.1%)	1	-	-
Yes	285831	455 (0.2%)	1.08	0.98-1.18	0.1
Horse raced on sealed track in last 60 days					
No*	3326677	4861 (0.1%)	1	-	-
Yes	524982	872 (0.2%)	1.14	1.06-1.22	<0.001
Horse raced on sealed track in last 180 days					
No*	2859434	4048 (0.1%)	1	-	-
Yes	992225	1685 (0.2%)	1.2	1.13-1.27	<0.001
Void Claim Rule in place at track					
None*	2202672	3538 (0.2%)	1	-	-
Type 1	199317	321 (0.2%)	1	0.89-1.12	1
Type 2	356635	518 (0.1%)	0.9	0.82-0.99	0.03
Type 3	279446	351 (0.1%)	0.78	0.7-0.87	<0.001
Type 4	529969	694 (0.1%)	0.82	0.75-0.88	<0.001
Type 5	283620	311 (0.1%)	0.68	0.61-0.76	<0.001
